# Supplementary material for: A Likelihood Approach for Real-Time Calibration of Stochastic Compartmental Epidemic Models
Source: PLoS Comput Biol. 2017 Jan 17;13(1):e1005257. doi: 10.1371/journal.pcbi.1005257 (PMC5240920; doi:10.1371/journal.pcbi.1005257)
Supplement: S1 File — (TAR.GZ) [file pcbi.1005257.s014.tar.gz › HSPH_Online-SI-Revision/MSS10/n10-severe/n10-severe_NEW2_table4.pdf]

1	newly 3 weeks spec	197.	283.4	{260., 309.}	43.8579	0.157097	newly 3 weeks cumul	743.	1125.9	{1087., 1167.}	51.5343	0.18034	newly 5 weeks spec	99.	153.74	{136., 172.}	55.2929	0.189405	newly 5 weeks cumul	972.	1489.18	{1433., 1544.}	53.2078	0.185098	newly 47 weeks cumul	1325.	1890.09	{1801., 1966.}	42.6483	0.154002
2	newly 3 weeks spec	183.	133.79	{115., 149.}	26.8907	0.138806	newly 3 weeks cumul	725.	556.99	{522., 594.}	23.1738	0.115125	newly 5 weeks spec	116.	70.32	{54., 85.}	39.3793	0.225004	newly 5 weeks cumul	1003.	724.71	{664., 791.}	27.7458	0.141934	newly 49 weeks cumul	1262.	897.83	{809., 986.}	28.8566	0.14897
3	newly 3 weeks spec	87.	72.27	{49., 94.}	22.2644	0.115379	newly 3 weeks cumul	412.	348.45	{287., 409.}	16.9005	0.0834618	newly 5 weeks spec	40.	29.42	{13., 47.}	36.7	0.221193	newly 5 weeks cumul	486.	423.97	{329., 503.}	15.9897	0.0783932	newly 51 weeks cumul	514.	477.83	{357., 584.}	15.0837	0.0709048
4	newly 3 weeks spec	158.	138.46	{115., 164.}	14.7342	0.0713842	newly 3 weeks cumul	634.	620.4	{564., 680.}	6.25237	0.0277052	newly 5 weeks spec	66.	63.97	{46., 83.}	15.7727	0.07248	newly 5 weeks cumul	805.	778.42	{699., 856.}	7.13043	0.0319052	newly 46 weeks cumul	946.	902.84	{793., 995.}	7.91755	0.0360287
5	newly 3 weeks spec	156.	75.05	{48., 100.}	51.891	0.338387	newly 3 weeks cumul	639.	368.66	{300., 429.}	42.3067	0.243879	newly 5 weeks spec	112.	30.44	{15., 45.}	72.8214	0.607432	newly 5 weeks cumul	896.	445.86	{351., 531.}	50.2388	0.309171	newly 46 weeks cumul	1167.	497.1	{386., 612.}	57.4036	0.377342
6	newly 3 weeks spec	63.	89.01	{57., 120.}	46.8413	0.162096	newly 3 weeks cumul	405.	437.32	{334., 537.}	16.7111	0.0695502	newly 5 weeks spec	51.	36.1	{13., 56.}	36.2745	0.245735	newly 5 weeks cumul	521.	529.61	{390., 643.}	15.8445	0.0702436	newly 47 weeks cumul	643.	589.47	{396., 776.}	18.1788	0.0889864
7	newly 3 weeks spec	115.	66.69	{45., 85.}	42.0087	0.24742	newly 3 weeks cumul	383.	273.93	{216., 328.}	28.4778	0.150594	newly 5 weeks spec	76.	35.45	{21., 51.}	53.3553	0.355443	newly 5 weeks cumul	548.	359.22	{279., 431.}	34.4489	0.188863	newly 46 weeks cumul	668.	451.71	{351., 546.}	32.3787	0.176182
8	newly 3 weeks spec	100.	63.96	{41., 85.}	37.04	0.216547	newly 3 weeks cumul	467.	315.3	{240., 391.}	32.4839	0.177159	newly 5 weeks spec	34.	25.03	{13., 40.}	34.6765	0.206484	newly 5 weeks cumul	561.	379.05	{276., 468.}	32.4367	0.177359	newly 50 weeks cumul	610.	418.52	{318., 502.}	31.4197	0.171203
9	newly 3 weeks spec	135.	118.72	{81., 148.}	17.6889	0.090484	newly 3 weeks cumul	764.	594.4	{494., 692.}	22.2225	0.11281	newly 5 weeks spec	57.	45.07	{23., 67.}	30.2632	0.17264	newly 5 weeks cumul	905.	713.57	{587., 846.}	21.1635	0.107797	newly 54 weeks cumul	1026.	786.7	{627., 934.}	23.3314	0.120807
10	newly 3 weeks spec	69.	63.49	{45., 83.}	20.6232	0.0978631	newly 3 weeks cumul	417.	343.27	{268., 415.}	19.1343	0.0964521	newly 5 weeks spec	31.	22.36	{10., 37.}	38.	0.232356	newly 5 weeks cumul	500.	402.24	{314., 492.}	21.12	0.1076	newly 54 weeks cumul	536.	432.36	{336., 511.}	21.1978	0.107584
11	newly 3 weeks spec	22.	10.61	{0., 27.}	66.0455	0.824803	newly 3 weeks cumul	176.	101.32	{32., 184.}	46.1023	0.353106	newly 5 weeks spec	7.	2.67	{0., 12.}	97.5714	1.4239	newly 5 weeks cumul	192.	108.67	{32., 193.}	46.6823	0.364949	newly 53 weeks cumul	192.	110.06	{32., 193.}	46.2083	0.361417
12	newly 3 weeks spec	117.	107.52	{79., 137.}	16.3932	0.0802486	newly 3 weeks cumul	568.	533.95	{460., 607.}	9.66373	0.0459133	newly 5 weeks spec	54.	41.07	{19., 63.}	31.3889	0.189541	newly 5 weeks cumul	704.	641.63	{540., 741.}	11.5781	0.0563682	newly 53 weeks cumul	804.	709.51	{578., 843.}	14.342	0.0709431
13	newly 3 weeks spec	134.	140.2	{118., 165.}	11.1045	0.0463347	newly 3 weeks cumul	648.	657.36	{603., 708.}	5.3179	0.0228137	newly 5 weeks spec	83.	60.57	{42., 77.}	27.9639	0.152944	newly 5 weeks cumul	810.	808.69	{740., 886.}	5.63333	0.0245637	newly 50 weeks cumul	934.	919.3	{828., 1026.}	6.97859	0.0308762
14	newly 3 weeks spec	60.	48.7	{23., 77.}	33.3333	0.194052	newly 3 weeks cumul	328.	267.4	{177., 356.}	22.7866	0.121114	newly 5 weeks spec	20.	16.39	{3., 29.}	44.45	0.339685	newly 5 weeks cumul	376.	312.54	{198., 419.}	23.2234	0.123318	newly 52 weeks cumul	397.	333.8	{201., 462.}	24.	0.12705
15	newly 3 weeks spec	54.	75.38	{47., 112.}	45.2222	0.150289	newly 3 weeks cumul	371.	416.78	{305., 549.}	22.2049	0.0867005	newly 5 weeks spec	11.	24.59	{11., 39.}	125.909	0.31776	newly 5 weeks cumul	406.	484.5	{349., 653.}	26.3596	0.0977536	newly 56 weeks cumul	423.	517.03	{365., 704.}	28.8771	0.105545
16	newly 3 weeks spec	69.	50.56	{35., 64.}	27.6522	0.150125	newly 3 weeks cumul	368.	239.07	{199., 270.}	35.0353	0.190556	newly 5 weeks spec	29.	21.94	{12., 32.}	29.4483	0.17185	newly 5 weeks cumul	440.	293.22	{242., 337.}	33.3591	0.179462	newly 52 weeks cumul	501.	334.85	{273., 393.}	33.1637	0.17912
17	newly 3 weeks spec	221.	115.09	{85., 145.}	47.9231	0.29356	newly 3 weeks cumul	873.	547.62	{450., 632.}	37.2715	0.206544	newly 5 weeks spec	183.	47.61	{26., 67.}	73.9836	0.620288	newly 5 weeks cumul	1256.	671.18	{525., 792.}	46.5621	0.277223	newly 52 weeks cumul	1842.	753.49	{562., 894.}	59.0939	0.394468
18	newly 3 weeks spec	189.	193.86	{170., 218.}	7.86243	0.0333837	newly 3 weeks cumul	833.	815.49	{762., 866.}	4.01561	0.0178282	newly 5 weeks spec	133.	95.9	{76., 113.}	27.8947	0.147282	newly 5 weeks cumul	1151.	1049.44	{988., 1117.}	8.85665	0.0407509	newly 50 weeks cumul	1580.	1273.85	{1184., 1365.}	19.3766	0.0941453
19	newly 3 weeks spec	134.	192.5	{172., 213.}	43.6567	0.155904	newly 3 weeks cumul	645.	723.24	{688., 755.}	12.1302	0.0494305	newly 5 weeks spec	78.	120.99	{106., 138.}	55.1154	0.187791	newly 5 weeks cumul	830.	996.95	{948., 1049.}	20.1145	0.0792742	newly 46 weeks cumul	1044.	1415.05	{1317., 1509.}	35.5412	0.13149
20	newly 3 weeks spec	271.	134.93	{72., 168.}	50.2103	0.334945	newly 3 weeks cumul	966.	564.43	{474., 642.}	41.5704	0.240734	newly 5 weeks spec	160.	76.91	{20., 107.}	51.9313	0.433969	newly 5 weeks cumul	1369.	742.97	{560., 854.}	45.729	0.276173	newly 46 weeks cumul	1954.	959.77	{576., 1171.}	50.8818	0.326301
21	newly 3 weeks spec	92.	60.76	{45., 80.}	34.0652	0.192101	newly 3 weeks cumul	451.	330.22	{286., 387.}	26.7805	0.138676	newly 5 weeks spec	39.	22.14	{10., 34.}	44.359	0.296885	newly 5 weeks cumul	575.	388.87	{324., 455.}	32.3704	0.173568	newly 54 weeks cumul	717.	417.51	{343., 488.}	41.7699	0.239287
22	newly 3 weeks spec	126.	140.94	{105., 182.}	24.3651	0.117235	newly 3 weeks cumul	547.	607.88	{532., 712.}	17.1627	0.0739576	newly 5 weeks spec	54.	68.2	{43., 95.}	42.8519	0.282769	newly 5 weeks cumul	674.	774.35	{669., 914.}	22.1083	0.094992	newly 52 weeks cumul	743.	919.21	{778., 1091.}	31.3553	0.128209
23	newly 3 weeks spec	180.	102.92	{74., 132.}	42.8222	0.252309	newly 3 weeks cumul	675.	447.81	{379., 525.}	33.6578	0.181794	newly 5 weeks spec	110.	46.66	{28., 68.}	57.5818	0.394231	newly 5 weeks cumul	921.	565.99	{466., 667.}	38.5461	0.215469	newly 51 weeks cumul	1186.	668.45	{546., 773.}	43.6383	0.253351
24	newly 3 weeks spec	117.	100.25	{82., 120.}	16.1453	0.0794253	newly 3 weeks cumul	561.	534.07	{474., 586.}	7.19964	0.0328903	newly 5 weeks spec	36.	34.65	{20., 49.}	25.5278	0.122267	newly 5 weeks cumul	658.	628.01	{554., 695.}	7.29939	0.0334041	newly 52 weeks cumul	737.	676.01	{577., 751.}	9.58887	0.0450709
25	newly 3 weeks spec	33.	17.86	{5., 33.}	52.6061	0.433676	newly 3 weeks cumul	182.	148.6	{72., 222.}	29.8571	0.180548	newly 5 weeks spec	2.	4.23	{0., 12.}	193.5	0.754347	newly 5 weeks cumul	198.	161.25	{79., 239.}	30.0253	0.182719	newly 55 weeks cumul	198.	163.72	{79., 239.}	30.3232	0.182494
26	newly 3 weeks spec	84.	92.27	{60., 127.}	25.3452	0.10584	newly 3 weeks cumul	510.	435.74	{351., 525.}	16.7255	0.0832148	newly 5 weeks spec	55.	39.87	{22., 59.}	32.3091	0.193131	newly 5 weeks cumul	628.	538.37	{416., 659.}	17.0334	0.0854266	newly 54 weeks cumul	701.	608.01	{451., 730.}	16.2325	0.0814765
27	newly 3 weeks spec	183.	130.69	{103., 158.}	28.6721	0.153219	newly 3 weeks cumul	804.	650.9	{563., 738.}	19.2015	0.0951264	newly 5 weeks spec	88.	48.74	{33., 64.}	44.6136	0.272698	newly 5 weeks cumul	1035.	782.1	{664., 906.}	24.4348	0.124517	newly 55 weeks cumul	1231.	856.04	{720., 1009.}	30.4598	0.160955
28	newly 3 weeks spec	70.	61.68	{40., 87.}	26.2286	0.131576	newly 3 weeks cumul	412.	349.37	{246., 450.}	20.0947	0.102054	newly 5 weeks spec	15.	17.94	{5., 30.}	52.9333	0.245881	newly 5 weeks cumul	458.	400.88	{288., 525.}	19.6026	0.0973218	newly 54 weeks cumul	486.	425.92	{306., 570.}	20.07	0.099949
29	newly 3 weeks spec	27.	38.01	{17., 57.}	58.1111	0.206757	newly 3 weeks cumul	254.	255.53	{192., 325.}	17.0591	0.0763958	newly 5 weeks spec	11.	9.42	{1., 18.}	54.5455	0.433382	newly 5 weeks cumul	278.	285.59	{211., 375.}	18.723	0.0819382	newly 56 weeks cumul	279.	296.87	{211., 392.}	20.9211	0.0885973
30	newly 3 weeks spec	46.	48.55	{29., 68.}	27.1957	0.115398	newly 3 weeks cumul	317.	327.46	{249., 397.}	14.795	0.0625462	newly 5 weeks spec	3.	12.66	{4., 23.}	328.	0.576831	newly 5 weeks cumul	342.	363.57	{269., 446.}	16.5702	0.0682098	newly 56 weeks cumul	343.	375.6	{281., 461.}	18.0758	0.0724636
31	newly 3 weeks spec	146.	67.11	{31., 100.}	54.0342	0.434072	newly 3 weeks cumul	569.	342.47	{223., 435.}	39.9701	0.246251	newly 5 weeks spec	41.	24.93	{0., 46.}	45.878	0.516285	newly 5 weeks cumul	686.	408.05	{260., 542.}	40.5379	0.255812	newly 53 weeks cumul	786.	446.56	{288., 600.}	43.1858	0.279459
32	newly 3 weeks spec	142.	89.16	{70., 110.}	37.2113	0.209612	newly 3 weeks cumul	533.	345.38	{296., 393.}	35.2008	0.190931	newly 5 weeks spec	69.	50.79	{30., 69.}	28.8551	0.162333	newly 5 weeks cumul	702.	462.65	{387., 549.}	34.0954	0.184584	newly 46 weeks cumul	971.	618.38	{490., 763.}	36.3151	0.201683
33	newly 3 weeks spec	184.	124.66	{104., 149.}	32.25	0.17338	newly 3 weeks cumul	700.	487.56	{435., 528.}	30.3486	0.158408	newly 5 weeks spec	80.	69.02	{50., 91.}	19.425	0.0971162	newly 5 weeks cumul	923.	648.01	{579., 714.}	29.7931	0.155237	newly 46 weeks cumul	1232.	844.87	{728., 958.}	31.4229	0.166192
34	newly 3 weeks spec	186.	268.71	{247., 289.}	44.4677	0.158946	newly 3 weeks cumul	603.	933.24	{903., 962.}	54.7662	0.189534	newly 5 weeks spec	102.	195.13	{171., 215.}	91.3039	0.28026	newly 5 weeks cumul	831.	1360.53	{1313., 1407.}	63.722	0.213944	newly 46 weeks cumul	1130.	2263.46	{2161., 2368.}	100.306	0.301459
35	newly 3 weeks spec	127.	64.24	{37., 96.}	49.9055	0.334158	newly 3 weeks cumul	520.	361.99	{265., 471.}	31.1096	0.172303	newly 5 weeks spec	58.	19.4	{5., 32.}	66.8276	0.573029	newly 5 weeks cumul	660.	418.64	{305., 523.}	37.0636	0.213046	newly 53 weeks cumul	785.	445.88	{318., 581.}	43.335	0.261621
36	newly 3 weeks spec	227.	208.44	{181., 233.}	9.60352	0.0449246	newly 3 weeks cumul	850.	823.11	{767., 874.}	4.66	0.020917	newly 5 weeks spec	131.	116.39	{94., 139.}	13.9924	0.0674867	newly 5 weeks cumul	1137.	1097.33	{1015., 1170.}	4.95602	0.0222928	newly 49 weeks cumul	1502.	1414.57	{1279., 1541.}	7.02197	0.0322754
37	newly 3 weeks spec	162.	133.72	{99., 166.}	19.4691	0.0997008	newly 3 weeks cumul	656.	567.55	{473., 649.}	14.1692	0.0689597	newly 5 weeks spec	79.	64.38	{44., 85.}	24.0253	0.128448	newly 5 weeks cumul	838.	726.84	{604., 828.}	14.2387	0.0696232	newly 52 weeks cumul	1008.	866.5	{686., 1001.}	15.3929	0.0758294
38	newly 3 weeks spec	199.	133.89	{114., 157.}	32.7186	0.175989	newly 3 weeks cumul	840.	592.76	{529., 649.}	29.4333	0.152723	newly 5 weeks spec	85.	60.18	{43., 78.}	29.3412	0.161085	newly 5 weeks cumul	1088.	742.41	{669., 827.}	31.7638	0.167474	newly 52 weeks cumul	1378.	864.64	{752., 1000.}	37.254	0.204371
39	newly 3 weeks spec	207.	138.3	{110., 160.}	33.1884	0.179954	newly 3 weeks cumul	748.	539.98	{468., 596.}	27.8102	0.143182	newly 5 weeks spec	130.	78.52	{63., 95.}	39.6	0.225789	newly 5 weeks cumul	1035.	721.95	{632., 796.}	30.2464	0.158083	newly 48 weeks cumul	1546.	943.08	{807., 1054.}	38.9987	0.216589
40	newly 3 weeks spec	211.	167.99	{144., 193.}	20.3934	0.10178	newly 3 weeks cumul	819.	689.4	{632., 753.}	15.8242	0.0756081	newly 5 weeks spec	116.	90.19	{72., 108.}	22.5086	0.115668	newly 5 weeks cumul	1057.	902.44	{830., 972.}	14.6225	0.0694689	newly 48 weeks cumul	1430.	1121.33	{1024., 1223.}	21.5853	0.106674
41	newly 3 weeks spec	77.	65.69	{39., 89.}	23.8831	0.12486	newly 3 weeks cumul	495.	398.25	{308., 500.}	21.0768	0.108881	newly 5 weeks spec	16.	20.03	{9., 34.}	47.3125	0.17806	newly 5 weeks cumul	559.	454.3	{346., 569.}	20.6691	0.106699	newly 56 weeks cumul	602.	478.36	{348., 613.}	22.5349	0.118189
42	newly 3 weeks spec	107.	169.7	{149., 193.}	58.5981	0.198211	newly 3 weeks cumul	500.	746.5	{691., 801.}	49.3	0.173481	newly 5 weeks spec	44.	80.89	{65., 95.}	83.8409	0.259621	newly 5 weeks cumul	615.	943.08	{877., 1008.}	53.3463	0.185053	newly 47 weeks cumul	696.	1110.85	{1018., 1193.}	59.6049	0.202321
43	newly 3 weeks spec	71.	60.	{43., 78.}	20.9859	0.108083	newly 3 weeks cumul	380.	340.76	{260., 402.}	12.8158	0.0623065	newly 5 weeks spec	40.	19.82	{9., 32.}	50.5	0.357724	newly 5 weeks cumul	453.	395.11	{303., 463.}	14.5453	0.0717291	newly 55 weeks cumul	496.	425.3	{315., 496.}	16.1169	0.0800459
44	newly 3 weeks spec	171.	102.35	{73., 141.}	40.3801	0.237962	newly 3 weeks cumul	617.	450.07	{362., 561.}	27.269	0.143943	newly 5 weeks spec	118.	47.88	{26., 68.}	59.4237	0.421568	newly 5 weeks cumul	869.	569.4	{456., 718.}	34.5316	0.190798	newly 49 weeks cumul	1172.	670.84	{498., 848.}	42.7679	0.251901
45	newly 3 weeks spec	123.	140.3	{118., 163.}	16.1138	0.0629677	newly 3 weeks cumul	569.	669.78	{612., 727.}	17.7153	0.0699394	newly 5 weeks spec	45.	59.4	{43., 74.}	34.2667	0.121671	newly 5 weeks cumul	691.	820.13	{740., 893.}	18.6874	0.0734337	newly 52 weeks cumul	789.	922.48	{828., 1017.}	16.9404	0.0667589
46	newly 3 weeks spec	168.	127.6	{95., 166.}	25.3571	0.138288	newly 3 weeks cumul	709.	537.18	{442., 647.}	24.7757	0.127476	newly 5 weeks spec	88.	61.	{42., 84.}	32.1818	0.227431	newly 5 weeks cumul	909.	686.1	{556., 821.}	24.9901	0.129852	newly 51 weeks cumul	1016.	824.1	{648., 996.}	20.0374	0.102863
47	newly 3 weeks spec	173.	123.18	{87., 154.}	29.3064	0.15948	newly 3 weeks cumul	651.	543.43	{440., 641.}	17.1905	0.0854577	newly 5 weeks spec	105.	57.51	{37., 83.}	45.2286	0.281261	newly 5 weeks cumul	904.	686.33	{555., 830.}	24.1051	0.124273	newly 52 weeks cumul	1045.	802.89	{637., 1002.}	23.6392	0.122329
48	newly 3 weeks spec	241.	145.71	{120., 174.}	39.5394	0.222564	newly 3 weeks cumul	934.	644.24	{578., 708.}	31.0236	0.162624	newly 5 weeks spec	108.	67.72	{51., 85.}	37.2963	0.212561	newly 5 weeks cumul	1207.	810.53	{728., 886.}	32.8476	0.17449	newly 49 weeks cumul	1531.	946.97	{839., 1057.}	38.147	0.210479
49	newly 3 weeks spec	134.	106.91	{74., 138.}	22.2313	0.121433	newly 3 weeks cumul	470.	486.19	{379., 565.}	13.3766	0.057894	newly 5 weeks spec	57.	47.87	{25., 75.}	30.5789	0.170134	newly 5 weeks cumul	619.	606.16	{454., 721.}	12.6914	0.0585335	newly 51 weeks cumul	792.	704.25	{496., 859.}	15.9154	0.0798209
50	newly 3 weeks spec	82.	72.34	{51., 92.}	17.	0.0862483	newly 3 weeks cumul	351.	400.19	{346., 442.}	16.1396	0.0648096	newly 5 weeks spec	30.	26.03	{15., 37.}	26.8333	0.140848	newly 5 weeks cumul	410.	469.76	{405., 534.}	16.839	0.0673409	newly 56 weeks cumul	413.	506.17	{442., 583.}	24.0363	0.0923383
